# Postpartum Psychosis as a Consequence of Thyroiditis Versus Relapse: A Diagnostic Dilemma

**DOI:** 10.7759/cureus.52357

**Published:** 2024-01-16

**Authors:** Alec Fulton, Neha Mittal, Anasua Deb

**Affiliations:** 1 Internal Medicine, Texas Tech University Health Sciences Center, Lubbock, USA

**Keywords:** postpartum psychosis risk factors, management of thyrotoxicosis, diagnostic dilema, postpartum psychosis, postpartum thyroiditis

## Abstract

Thyrotoxicosis can exhibit overlapping symptoms of psychosis in the general population. Each of these pathologies has well-established workups and management. Rare presentations of thyroiditis and psychosis in the postpartum state have been seen in case studies mostly, but data on the prevalence of postpartum psychosis in association with postpartum thyroiditis are not available. Here, we present a unique case of a patient with a history of bipolar disorder who originally presented with postpartum thyroiditis that was worked up and managed appropriately. However, on follow-up, the patient was found to have progressed into prominent psychosis. Both thyroiditis and psychosis were managed individually with full remission upon discharge and is doing well today. The co-occurrence of postpartum psychosis and thyroiditis presents a unique challenge for timely diagnosis and management. We present a case of a young woman initially diagnosed with postpartum thyroiditis needing further management of postpartum psychosis due to persistent symptoms. Clinical presentation supported with a prior history of mood disorder increases the likelihood of these diagnoses together.

## Introduction

Postpartum thyroiditis and postpartum psychosis are often both considered in the differentials of patients presenting with postpartum psychosis and become primary diagnoses only after medical conditions are ruled out [1]. However, unique cases have been reported of the co-occurrence of these pathologies in postpartum women and successfully treated using antipsychotics loxapine and amoxapine for psychosis alongside propanol and propylthiouracil for thyroiditis [2]. Although this presentation has been recorded, the prevalence of postpartum psychosis is thought to be around 0.1-2% [3], and the prevalence of psychosis co-occurring with thyroiditis in the postpartum state has not been well established. With little established data on this co-occurrence, it makes sense why patients with postpartum thyroiditis may not raise suspicion with clinicians for concurrent psychosis. In this case, our patient presented with features that could all be attributed to hyperthyroidism and thought to be purely thyrotoxic symptoms, later on revealing severe symptoms of psychosis. We make the case that clinicians should have a higher suspicion of psychosis when seeing a patient with apparent thyroiditis in the postpartum period. Additionally, we believe it is important to treat these pathologies as separate for the best care of both the mother and infant.

## Case presentation

Initial encounter

A 35-year-old female, who was 10 weeks postpartum after twin delivery through a C-section, came in for a wellness (via telemedicine) check. Her recovery was complicated by her baby's stay in the neonatal intensive care unit (two weeks and five days, respectively), sleep deprivation, and grief associated with her uncle's hospitalization. During her visit, the patient reported that her babies were doing well; she had improved adjustment to them and a supportive family. Since returning to work two weeks before her visit, however, she reported increased stress and anxiety. The patient expressed specific symptoms including irritability, restlessness, and anxiety and mentioned that she had been seeking professional counseling to address these concerns. Her husband and coworkers noted the patient to have more pressured speech with tangential and disorganized thought processes, and during the visit, this presented as conversational jumping from nutritional concerns, home life anxiety, and work status. The patient also revealed other noteworthy symptoms of feeling hot constantly, tachycardia at rest measured on her Apple watch, and diarrhea that was temporally associated with breastfeeding.

Patient history 

The patient’s past medical history was significant for major depressive disorder diagnosed at the age of 18 years and treated with escitalopram for about two to three years. For subsequent diagnosis of type 1 bipolar disorder at age 23, she took valproate for two years. She decided to end pharmacologic therapy when she remained symptom-free for an extended time. Since then, the patient has reported having a few episodes of 1-2-week periods of decreased mood, but no other symptoms of mood disorder despite the stress of moving across countries in the middle of a global pandemic, residency training, and twin pregnancy. Her family history was notable for unspecified hypothyroidism in both of her parents and Crohn’s disease in her mother. She denied tobacco, alcohol, or illicit drug use. Her current medications included a prenatal vitamin supplement and 25 mcg of levothyroxine daily, prescribed by her reproductive endocrinologist before pregnancy and throughout gestation. Levothyroxine is commonly given to women desiring to conceive with elevated TSH because of studies on thyroid function and its effects on pregnancy and fetal development. These studies found increased rates of spontaneous miscarriage in women who had elevated TSH of 2.5 mIU/L or greater and, therefore, recommend achieving a target TSH of less than 2.5 mIU/L before and during pregnancy [4,5]. She remained euthyroid throughout her pregnancy with this dose and remained on the medication until the day of her presentation. Her pregnancy complications included gestational diabetes, which she controlled with diet and glyburide. Lab work showed serum free T4 of 3.05 ng/dL (n=0.93-1.70) and free T3 of 7.05 ng/dL (n=2.3-4.2). TSH was decreased at 0.01 mIU/I (n=0.27-4.2). Given her clinical symptoms and lab data, she was diagnosed with postpartum thyroiditis, and levothyroxine was discontinued. Serum anti-thyroid peroxidase antibodies (TPO-Ab) and thyroid-stimulating antibodies (TSA) were within normal range.

Hospitalization 

**Figure 1:**
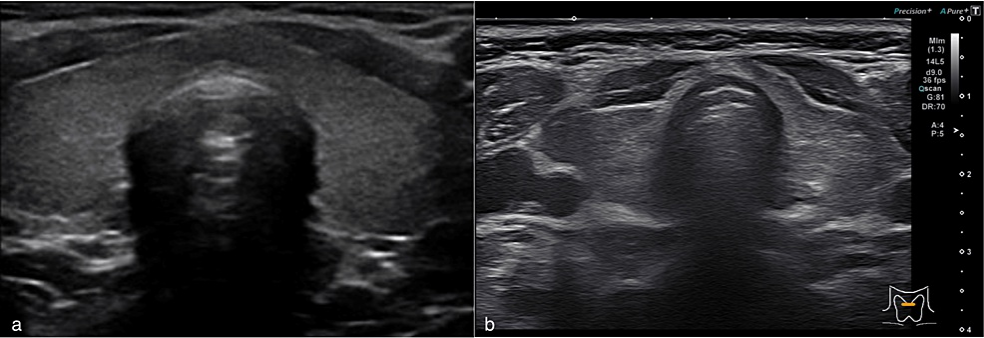
a) Ultrasound of normal thyroid gland with homogeneity. b) Ultrasound of our patient's thyroid gland showing evidence of slight heterogeneity. a): Source Ref. [6]

At one week follow-up, the patient expressed a variety of new worries for her and her babies’ safety, expressing delusional beliefs of being followed and watched by a criminal organization, irritability with family members, and anxiety that a hospital employee was feeding information about her to the criminal organization. Her husband confirmed the history but did not endorse her delusional thoughts as being true. The patient was hospitalized for symptoms consistent with mania with paranoia. On admission to the hospital, a repeat blood work showed mildly less elevated levels of free T3 (6.42) and free T4 (2.81). Ultrasound of the thyroid showed slight heterogeneity with no areas of increased vascularity or nodules present (Figure 1). Radioactive Iodine uptake was not done, as the patient was breastfeeding. After a psychiatric assessment, she was prescribed 20 mg of propranolol BID and 5 mg of olanzapine nightly. Her symptoms of mania, restlessness, and tachycardia began to improve by the third day of hospitalization, and she was discharged with the diagnosis of postpartum thyroiditis and postpartum psychosis.

Follow-up

**Figure 2:**
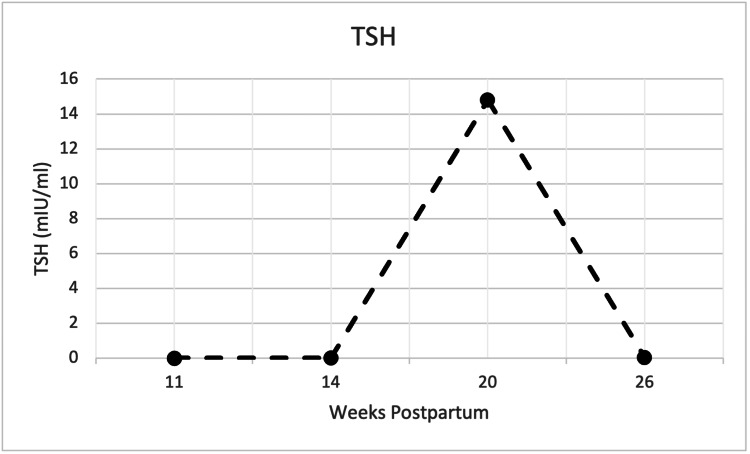
Serum TSH (mIU/mL) at postpartum weeks 11, 14, 20, and 26.

**Figure 3:**
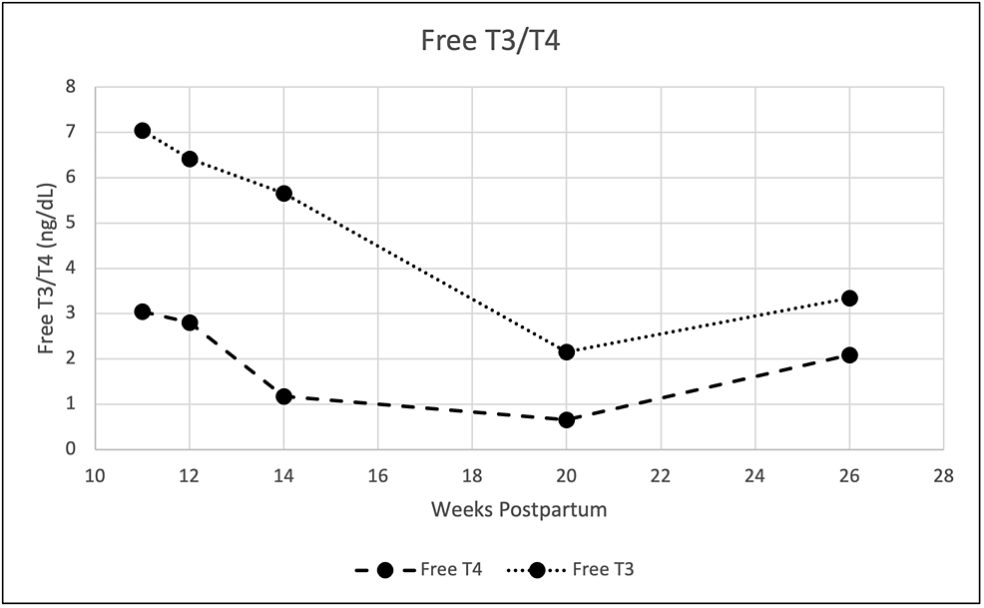
Serum free T3 and free T4 (ng/dL) at postpartum weeks 11-26.

Two weeks after the discharge, low TSH (Figure 2) and elevated free thyroid hormone levels (Figure 3) persisted. However, she reported improvement in symptoms from the time of hospitalization. In continual follow-ups with the patient, eight weeks post-hospitalization (20 weeks postpartum), the patient reported symptoms of cold intolerance, excess fatigue, loss of interest, and mild anxiety. At this time, the thyroid labs showed elevated TSH (14.8 mIU/mL) and reduced free T3/T4 indicating a hypothyroid state. The patient was prescribed levothyroxine 100 μg daily for hypothyroid state and with improvement of fatigue and energy, serum levels for anti-TPO antibodies remained normal.

## Discussion

Postpartum thyroiditis occurs in 5-8% [7] of women within the first 12 months of delivery, more commonly in women with positive anti-TPO antibody titers. The cause is hypothesized to be a rebound of immune activity after delivery in patients with underlying thyroid autoimmunity; the pathology of postpartum thyroiditis presents with findings similar to Hashimoto’s thyroiditis, and women commonly test positive for anti-TPO antibodies. Anti-thyroglobulin antibodies, a marker of destructive thyroiditis can be positive as well [8]. Approximately 50% of patients with postpartum thyroiditis will present with an isolated hypothyroid state, in about 25% of patients the course of the disease will start with a thyrotoxic phase within the first four months, which is then followed by a transient hypothyroid state lasting four to six months. The remaining 25% will present with only a thyrotoxic state followed by a return to the euthyroid state [9]. The timeline when our patient began displaying symptoms was within the expected time frame for postpartum thyroiditis. A negative serum thyroid stimulation (TSI) antibody ruled out the potential for Grave’s disease, which may have similar presenting symptoms [10]. A radioactive iodine uptake scan is another potential way for differentiating from Grave’s disease; however, this was opted against because of the patient’s breastfeeding status. Management of thyrotoxic symptoms, specifically tachycardia, can be accomplished with a beta-blocker such as propranolol, which should be slowly tapered off as the thyrotoxic state dissipates. Follow-up serum TSH should be taken annually in these patients to monitor for persistent hypothyroid state [11]. Postpartum thyroiditis is often transient; however, persistent hypothyroidism may follow. Significant predictors for the chronic hypothyroid state include continual elevations in anti-TPO antibodies as well as findings of hypoechogenicity on ultrasonography [2,12]. While irritability and hyperexcitability are typically seen in hyperthyroidism, symptoms of mania and psychosis are rare [13]. Few cases were reported, where postpartum psychosis presented with a hyperthyroid state from postpartum thyroiditis with elevated anti-TPO antibodies [14].

Postpartum psychosis, on the other hand, is a mood disorder occurring typically one to two weeks after delivery but can occur further out. It presents with a variety of symptoms including mania, anxiety, depression, and mood lability. Hallucinations and delusions are common and are typically centered on the newborn. Delusional symptoms are often associated with increased protective behavior or increased thoughts of harm to the newborn, although infanticide is rare, accounting for 1-4% of cases [15]. Postpartum psychosis has been reported to occur in about 0.1-0.2% of deliveries but at a much higher rate of about 20% in women with a history of bipolar disorder [3]. After treatment, the recovery from postpartum psychosis is good, but women who intend on having more children in the future have been found to have a 50% risk of recurrence of perinatal mood episodes [16]. Postpartum psychosis is managed per the guidelines in the general population. Our patient responded well to treatment with propranolol and olanzapine and continued to take the latter even when she was in a hypothyroid state. It is reasonable to consider managing both physical and psychiatric symptoms when the etiology is not as clear. Our case here presents a unique challenge of diagnosing a combination of both thyroiditis and bipolar depression in a woman whose psychosis symptoms relapsed in this setting of increased stress when she returned to work. Without this trigger, it is possible that her psychosis could have remained undiagnosed because of overlapping symptoms.

A retrospective study of thyrotoxic patients presenting with acute psychosis suggested a correlation between the two [17]. However, the prevalence of the same in the postpartum state has not been assessed, and rare publications mostly consisting of case reports are available. Few cases of similar presentation are reported in women with positive anti-TPO titers; our case does not show any evidence of thyroid autoimmunity. Some proposed ideas on the co-occurrence of psychosis and thyroiditis in the postpartum period include postpartum psychosis because of thyrotoxicosis caused by thyroiditis, precipitation of psychosis from thyroiditis in women with preexisting mood disorders, and coincidence of both thyroiditis and psychosis in the early postpartum period [2]. Over the years, an association between postpartum psychosis and autoimmune thyroid dysfunction with elevated anti-TPO levels has been reported. A recent case-control study recognized this pattern and showed that women with postpartum psychosis with no prior psychiatric history are two to four times as likely to be found to also have an autoimmune thyroid disease when compared to postpartum women with no psychosis [18]. Although correlations have been made between thyrotoxicosis and psychosis, causation has not been described with clarity. Life stressors, by itself, can trigger a relapse of bipolar episodes. Hence, it can be a challenge to establish a clear cause and effect.

## Conclusions

Our case is a rare presentation of postpartum thyroiditis and postpartum psychosis with no autoimmune thyroid dysfunction. Clinicians should consider the possibility of postpartum psychosis in patients with thyrotoxicosis particularly in high-risk individuals such as those with prior history of bipolar disorder and autoimmune thyroid dysfunction for timely management. In either case, whether psychosis follows a thyrotoxic state or occurs independently as a relapse in the postpartum state, simultaneous symptom management of both conditions is appropriate. Further research into clear definitions and management guidelines will assist physicians in taking care of these vulnerable patients. For patients with postpartum thyroiditis, continual follow-up for thyroid autoimmunity to monitor for progression or resolution of thyroid dysfunction is recommended. For postpartum psychosis, follow-up with a psychiatrist to monitor symptom resolution and management of any recurring symptoms is recommended as well. These patients should be counseled about the increased risk of relapse in the future postpartum state.
